# Bi-dimensional values and attitudes toward online fast food-buying intention during the COVID-19 pandemic: An application of VAB model

**DOI:** 10.3389/fnut.2022.894765

**Published:** 2022-11-25

**Authors:** Chen Yan, Abu Bakkar Siddik, Mohammad Masukujjaman, Qianli Dong, Muhammad Hamayun, Zheng Guang-Wen, Abdullah Mohammed Ibrahim

**Affiliations:** ^1^School of Economics and Management, Chang'an University, Xi'an, China; ^2^School of Management, University of Science and Technology of China (USTC), Hefei, China; ^3^Department of Business Administration, Northern University Bangladesh, Dhaka, Bangladesh; ^4^Department of Management Science and Commerce, Bacha Khan University, Charsadda, Pakistan; ^5^School of Economics and Management, Shaanxi University of Science and Technology (SUST), Xi'an, China

**Keywords:** fast food, millennial, value-attitude-behavior model, online buying intention, COVID-19

## Abstract

The purpose of the study is to determine the factors of online fast food-buying intention among Bangladeshi Millennials during the COVID-19 pandemic. The study adopted the Value-Attitude-Behavior (VAB) model and designed it as a higher-order constructs model to predict buying intention. Using a quantitative method (i.e., cross-sectional survey), data was collected from 325 respondents via a structured questionnaire and subsequently analyzed using Structural Equation Modeling (SEM) through AMOS software. The findings of the study revealed that convenience and food quality generate utilitarian values, while subjective norms and novelty-seeking form hedonic values. Also, utilitarian and hedonic values significantly affect cognitive and affective attitudes. As opposed to food quality, the cognitive attitude, affective attitude, self-identity, and subjective norms were observed to affect behavioral intention, with affective attitude producing the strongest association, albeit with the high explanatory power of the model. Consequently, this study offers a number of theoretical and policy implications to design better interventions that address public health regarding fast food consumption.

## Introduction

The ongoing COVID-19 pandemic has ushered in a new era in global consumption behavior (1). Consumers' behavior including their food-eating and buying pattern has been altered as a result of the pandemic (2). The enactment of lockdown to curb the spread of the COVID-19 virus restricted people's access to the grocery store, thereby generating concerns about food security and panic purchasing (3). Moreover, consumers' interest in the consumption of ultra-processed food seems to have risen during the lockdown and quarantine period, as these people can spend more time on several communication platforms such as computers, tablets, phones, and television through which information about this processed food may have been propagated (4). In keeping up with the new demand, producers and retailers of packaged products face substantial difficulties in terms of sales (drop in consumption, ensuring high hygiene environment and longer operating hours), marketing (need to involve with multiple platforms, win in loyalty shifts) and assortment (polarization in pack size-large and single packs-and hygiene certainty, rethinking brand mix) (5). On the other hand, the emergence of food delivery service providers back in 2013 in Bangladesh set the path for online food delivery, with the fast-food companies such as Pizza Hut, KFC, and Domino's pizza among others now availing delivery services to their customers via their website. However, the patronage of online food delivery services is likely to subside following the withdrawal of lockdown, as the majority of the customer can dine in physically. To attain long-term viability, service providers need to explore its capacity to meet and exceed the expectations of its current customer base (6). It has been observed that customers prefer to order meals using smartphone apps, websites, or social media due to being more convenient and faster. Consequently, analyzing important aspects impacting consumers' usage intention is critical in identifying factors favoring customers' preference for online fast-food purchases.

To date, academics have used a variety of theoretical models to investigate consumer attitudes toward online platforms, including the Theory of Planned Behavior (TPB), the Technology Acceptance Model (TAM), and the Unified Theory of Acceptance, Use, and Technology (UTAUT). Although different studies have utilized the Value-Attitude-Behavior (VAB) model in the studies of green purchase intention (7), green restaurant decisions (8), and internet memes (9), no research has used the VAB model in the investigation of fast food-buying intention to the best of our knowledge. VAB is a cognitive-behavioral model that shows how values, attitudes, and behaviors have a hierarchical effect, where the higher the level of value orientation, the higher the level of individual attitude, which in turn led to a higher level of behavioral intention and the actual behavior of individuals. The VAB model suggests the direct and indirect link of value and attitude in generating intention which is missing in the TPB and TAM models. Although TAM brought the attitude in such relations, the model is more suitable for technology adoption. The UTATUT model is the improved version of TPB and TAM, but it ignored considering the value and attitude in the model. Most people think that each customer has his or her own set of values that “reflect the choices an individual makes based on the different social values or value systems to which he or she is exposed” (10). In other words, value is a highly desirable quality that people use to make decisions and act in certain ways (11). An equally significant role in the formation of behavioral intentions and behavior is played by one's attitude. Moreover, the role of technology readiness in consumer behavior research has not received much attention in the existing literature. Hence, this research intends to close the gap by using the VAB model to determine the factors promoting online fast-food purchase.

Despite the rising popularity of online food buying, few empirical research has been conducted to determine the major factors influencing the desire to purchase food online. Existing literature on food buying have only covered the major factors affecting the acceptance/intention to purchase online (12, 13), products or sellers' characteristics affecting purchase intention (14, 15), and product information that lead to a higher online purchase (16). Other studies have looked into the online food buyer's behavior, and comparison between online and offline purchases (17, 18), the variables that lead to online buying (17, 19), and the impact of various situational circumstances on online food purchases (20). Although much research has been conducted on fast food-buying intention (21–26) and online fast-food delivery systems/apps (27–31), few research (32, 33) have investigated online fast food-buying intention. The existing research presented their portrayal of a fragmented view of adoption and those lack a holistic model.

Researchers have discovered a slew of variables that associate consumers' decisions to buy fast food online or not. Previous research revealed that attitude influenced behavior (34, 35). Also, it has been discovered in several research that customers acquire items and services for utilitarian and hedonistic reasons (36, 37). Utilitarian attributes are those that customers associate with a product's usefulness and ease of use. Hedonism deals with emotional and gratifying sensory experiences such as feelings and pleasure. Utilitarianism and hedonism are widely accepted as the underlying motivations for consumer purchase decisions, in line with past research (37). Although few studies (38–41) have attempted to employ bi-dimensional attitudes and values in various fields, their use in the study of fast food-buying intention is lacking to the best of the researcher's knowledge. Fast-food intake was expected to be affected by both factors, but only the cognitive attitude was shown to be a significant predictor of fast-food consumption (42). Further testing in diverse contexts (particularly online fast-food buying) is required to justify the predictability of emotional attitudes toward fast-food intake in this scenario. Besides, in the case of online fast food-buying intention, past research failed to incorporate these two-dimensional values and attitudes into a single model. The roles of attitude are not one-dimensional and it is needed to determine consumers' behavior in greater detail using cognitive and affective dimensions. Cognitive attitude is the liking or disliking based on the functions and utilities, whereas affective attitude is based on the emotional and sensational experience of objectives. Thus, some gaps prevail in the academia that seeks to address a comprehensive understanding of this sector.

In addressing the aforementioned gaps, this study develops and examines a holistic model by incorporating bi-dimensional values and attitudes incorporating higher-order construct format in the Bangladeshi context. The current study examined the factors related to online fast food-buying intention during the COVID-19 pandemic. The findings of this study will provide restaurants with a better understanding of their customers and the factors influencing their online fast-food purchases during and after the pandemic. This research expands the literature on online fast food purchasing by applying the VAB model and examining the determinants of online fast food purchasing in the context of emerging economies like Bangladesh during the COVID-19 pandemic. With this article, fast food providers will be able to prepare for future restrictions as well as the post-vaccination period by learning about the determinants of online purchase intention during the quarantine period.

## Literature review and hypothesis development

### Fast food and online food delivery in Bangladesh

Although “fast food” does not have a definite meaning, it generally refers to meals that can be supplied immediately and on-demand. This definition encompasses food such as sandwiches, hamburgers, fried chicken, fries, etc., supplied by KFC, AFC, Pizza Hut, A&W, Fortune Fried Chicken (FFC), and California Fried Chicken (CFC, McDonald's). Although fast food is often characterized by high calories, sugar, and fat, it remains a popular choice among consumers, since it is handy, flavorful, and reasonably inexpensive. According to Goyal and Singh (43), fast food outlets are the world's fastest-growing food sector, offering subscriptions for both dine-in and take-out experiences. Due to the hectic schedules of most working families, especially those with young children, fast food is often preferred to home-cooked food (44).

The online food delivery industry is a highly growing sector of Bangladesh. During the lockdown, there has been an increase in the number of customers (15–20%) looking for food to be delivered online because restaurants are only permitted to offer delivery to homes or takeaway services at a limited time (45). The delivery is operationalized in two ways; Platform-to- Consumer (user around 2.7 million) and Restaurant-to-Consumer delivery (User around 5.8 million) (46). It is anticipated that the total number of users will increase to 10.0 million by the year 2026, up from 7.5 million in 2022. Restaurant-to-Consumer Delivery is predicted to have the highest market volume in 2022, at US$79.45 m. This makes it the largest section of the market (46).

### The millennials

Generation Y people are sometimes called “Millennials,” “Generation Me,” or “Echo Boomers” (47–49) because they are the “baby boomers' offspring.” They are products of the high birth rates between the early 1980s and the mid-1990s (ages now between 26 and 41 years), very well-educated, and cannot be deceived by traditional marketing methods. In addition, they are more diverse in race and ethnicity, utilize different types of media, exhibit significant brand loyalty, and are more likely to adopt new habits, styles, and ways of communicating, thanks to their access to the internet (50).

Millennials are now a sizable demographic, and the consumer goods industry sees them as a promising demographic to target because of their disposable income. Since millennials' behavior differs so significantly from that of previous generations, studying them has taken on new significance and importance (51). They are distinguished in part because they will account for half of the world's consumption in 2017 (52). This generation group is more active than previous ones in incorporating technologies into their day-to-day lives for the purposes of marketing. They use their mobile devices and the traditional means of connecting to the Internet to connect to stores or brands (53). Small online stores have benefited from this important group of customers because of their ability to buy things and use technology (52).

### Theoretical framework

#### Value attitude behavior (VAB) model

**Figure 1:**
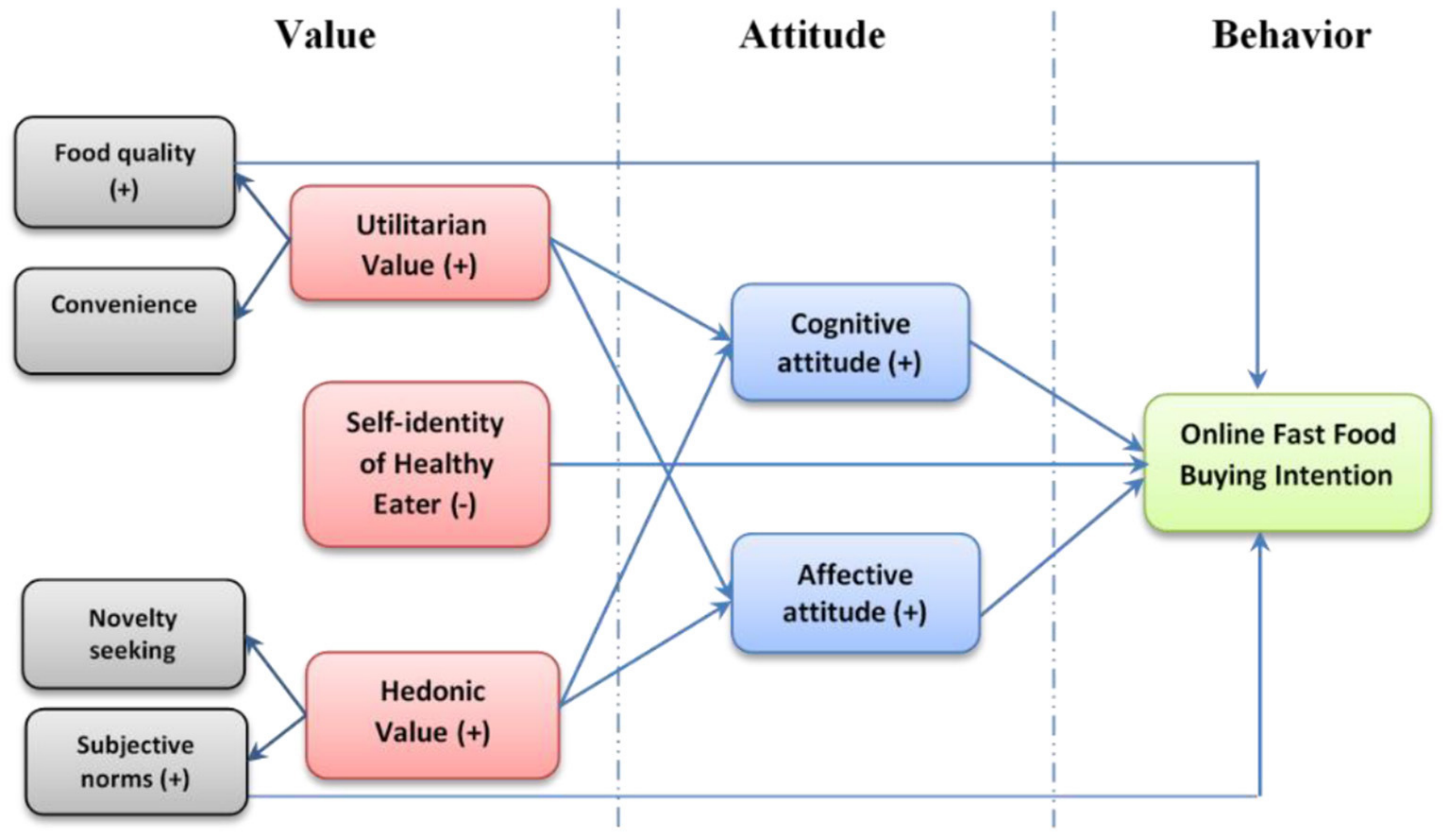
Conceptual framework using VAB model.

The VAB model is commonly used in social psychology to explore and understand behavior (54). Consumers' values are said to influence their attitude and behavior toward specific products (54, 55). According to Tudoran et al. (56) values influence behavior indirectly via attitudes. Hence, values, attitudes, and behaviors (VAB) are three factors that make up the framework of this study (Figure 1). According to the VAB paradigm, values are organized hierarchically and their perceptions impact customers' attitudes, which in turn drive their behaviors (57). Values are the most abstract social cognitions, according to Rajani (58), and are expressed in attitudes and behaviors. People's behaviors are guided by the notion of value, which is a desired and basic norm (59). Consumers' social and psychological growth will allow them to gradually create a sense of value that is more subjective and individualized in character. Therefore, it is crucial to consider an individual's mindset while determining their actions. The perceived worth of a person affects attitudes in both direct and indirect ways (56). Since attitudes and values are both based on abstract social cognitions, early research by Rokeach (60) argued that attitudes and values are more fundamentally related to attitudes, and behavior were arranged in a hierarchical sequence.

Values represent consumers' behavior by affecting the flow from abstract values to midrange attitudes to specific behaviors (i.e., the VAB hierarchy). Using the VAB paradigm, researchers have been able to study many consumer behaviors, including the purchase of organic goods (61), e-shopping (62), and the acceptance of mobile healthcare services (63). With the VAB model, Jayawardhena (62) explored internet shopping environments and found a link between good attitudes toward online buying and personal values like hedonic value and self-fulfillment. As a result, this association may be used to forecast customers' online fast food-purchasing habits. Therefore, the conceptual framework of the study can be shown in Figure 1.

### Hypothesis development

#### Utilitarian values

Regardless of whether they are buying in person or online, customers have a mix of utilitarian and hedonic interests (36, 64, 65). The concept of utilitarian value refers to a holistic evaluation of the advantages and disadvantages of various options (66, 67). Consumers expect items to perform as expected, and utilitarian value relates to whether that expectation is met. This includes economic values such as convenience, efficiency, and money (68). Lee and Yan (69, 70) found that utilitarian values of organic food, such as how consumers feel about its nutritional value, its effect on the environment, and its price, have a big impact on whether or not people are willing to buy it.

#### Convenience

Convenience is the length of time it takes to find a place, shop, product, and information about a product (63, 64). Rohm and Swaminathan (71) found that convenience has a major influence on online-purchasing behavior, which has resulted in the recent uptick in the popularity of online shopping. Convenience is an important factor in determining whether or not a customer will make an online purchase. Finding a product or business as well as information via the internet takes less time and effort, according to Childers and colleagues (72). Customers may place orders at any time and perform all their shopping in one location, thereby saving their time and gas. According to the findings of this research, customers benefit more from the higher utilitarian purchasing values provided by fast-food restaurants.

#### Food quality

In the fast-food sector, one of the most important aspects that determine purchase intention is the quality of the food served. Food quality, according to Sulek and Hensley (73), may include all features of a food captured in a single measure. Temperature, menu diversity, tastiness, and presentation are just a few of the factors that go into determining meal quality. Customers' purchasing decisions will be influenced favorably if the quality of their meals is improved. In light of the aforementioned, the following hypotheses are advanced:

***Hypothesis (H***_**1**_***):** The utilitarian value of consumers significantly and positively impacts their cognitive attitude*.

***Hypothesis (H***_**2**_***):** The utilitarian value of consumers significantly and positively impacts their affective attitude*.

***Hypothesis (H***_**3**_***):** The food quality significantly and positively affects their behavioral intention*.

#### Hedonic values

Hedonic value is becoming more and more important to customers (36), and according to Holbrook and Hirschman, it is the satisfaction customers feel after purchasing a product (74). In contrast to utilitarian values, hedonic values are more personal and subjective, resulting in a buying experience that includes fun, imagination, multisensory and emotional components of the product (75). The hedonic value of a product is based on the emotional or sentimental worth that customers gain from their purchase, mostly from the enjoyment and playfulness of the product. In certain circumstances, the experience of buying is more essential than the purchase itself (64). Relevant studies (76, 77) have concluded that a consumer's willingness to pay a premium price for organic food is justified by the pleasure and delight that the consumer derives from consuming organic food, as well as the consumer's interest in maintaining good health and a clean environment.

#### Novelty seeking

Consumers are motivated by a need for novelty, which is a sort of self-encouragement (78). According to Rishi and Mehra (79), people will be more willing to try duplicate products with a distinctive design or a design that closely resembles the original product. Cheap copy items and a wide range of original brand products make it easier for customers to satisfy their experimental and curiosities want (38). As a result of their pursuit of these goals, innovators seek to improve the perceived value of their products and services through the acquisition of new information and the introduction of novel elements (80). In addition, past research has shown that there is a favorable correlation between innovativeness and a person's intention to make use of new technologies (81, 82). Xue et al. (23) found that uniqueness seeking positively affects the purchase intention of fast food buying in Pakistan. As a result, consumers that enjoy trying something new will be more interested in fulfilling their internal drive by utilizing a new product.

#### Subjective norms

Along with hedonic advantages, websites' interactivity provides utilitarian advantages including saving time and energy, lowering the chance of errors, and increasing the number of better alternatives available to the user (83). Furthermore, consumers' opinions about online stores are thought to be improved by the interactivity of websites, which leads to an increase in website visits and online purchases (84, 85). These findings led us to believe that the hedonic and utilitarian benefits of website interaction may improve the attitude toward online shopping. Hence, the following assumptions are developed:

***Hypothesis (H4):** The hedonic value of consumers significantly and positively affects their cognitive attitude*.

***Hypothesis (H5):** The hedonic value of consumers significantly and positively affects their affective attitude*.

***Hypothesis (H6):** The subjective norms have significant and positive effects on their behavioral intention*.

#### Cognitive attitude and affective attitude

Having a positive or negative view toward someone, a location, or an item is known as an attitude (86). Assumptions, feelings, values, and consciousness are all components of attitude, which is not a one-dimensional entity (87). In accordance with Eroglu's (88) categorization of attitudes, we employed two categories of attitude in our research: cognitive attitude and emotional attitude. To have a positive or negative cognitive attitude toward anything, one must weigh the benefits and drawbacks of the object in question (87, 89). An affective attitude is concerned with the feelings and impressions a person has as a result of interacting with or being exposed to a certain thing (87). Attitude has been utilized in a variety of research and circumstances (86). There is a strong positive correlation between the mindset of customers and their purchasing habits (90). When it comes to internet buying, attitudes have been discovered to have a big impact on decision-making (86, 91). This reasoning led us to believe that cognitive and emotional attitudes and purchasing intentions are linked in a favorable and important way.

***Hypothesis (H7):** The cognitive attitude of consumers has significant and positive impacts on their purchase intention*.

***Hypothesis (H8):** The affective attitude of consumers has significant and positive impacts on their purchase intention*.

#### Self-identity of healthy eater

In their study on fashion buying behavior among generations, Valaei and Nikhashemi (92) proved a considerable correlation between self-identity and purchase intention. However, Salem and Chaichi (93) found no relationship between self-identification and behavioral intention. Past Studies in environmental psychology have looked at how individuals' self-identities might be used as a basis for forecasting their actions. Self-identity as a recycler predicts behaviors that lead to recycling (94), while self-identity as an environmental activist predicts behaviors that lead to activism (95). When it comes to food choice and consumption, self-identification has been proven to be a greater determinant of behavioral intention than other TPB components (96). This finding is also confirmed by other authors (42) who highlighted that those who consider themselves to be healthy eaters are less likely to eat regularly at fast-food restaurants. So, the greater the self-identification as a healthy eater, the lower the behavioral intention to buy fast food for individuals.

***Hypothesis (H9):** Self-identification as a healthy eater is negatively related to the behavioral intention to consume fast food*.

## Research methodology

This is an empirically based quantitative study. An original survey of consumers was used to get information about what was associated with consumers' decision to buy fast food online. Cross-sectional surveys were used, indicating that data was obtained to assess the population's inference at a certain point in time.

### Sample

The study population comprises young people (aged 26-41) who live in urban regions of Dhaka (Bangladesh) and consume fast food. In this investigation, we have adopted a non-probability sampling method, as opposed to probability sampling, due to the lack of consumer data repositories and sufficient resources. To utilize Structural Equation Modeling (SEM), a sample size of at least 200 is required, as suggested by Kline (97). However, some scholars have suggested that 5–10 replies per parameter is enough to get an appropriate sample size (98, 99). Therefore, a sample size of 299 or more is necessary to test the model using the above-mentioned criteria. This study took a sample size of 335 people to avoid prospective complexities arising from a limited sample size.

### Data collection process

The data was gathered by administering an online survey to fast-food consumers through Retailer Facebook Pages (RFP). Some of the most popular online fast-food merchants in Bangladesh are KFC, BFC, CFC, Pizza Hut, Domino's Pizza, CP Fried Chicken, American Burger, and Burger King. By using Facebook messaging between May and June 2021, we contacted around 500 customers, out of which 471 agreed to participate in the survey. Subsequently, the questionnaire URL was emailed to each one of them and a total of 125 responses was received in the first 2 weeks, while another 105 responses in the third week after a reminder. The remainder of the 325 required responses were received in the fourth and fifth weeks following a second reminder. Summarily, a total of 325 (65%) responses was obtained in the span of 5 weeks. To ensure our target group of young people, we requested only those who were in the target age range at the time of sending questionnaires. However, the collected data were screened out again and sorted out 30 responses that are beyond our age ranges. So, after the screen-out process, we finalize 335 responses for this study. During the collection of data, the permission of each respondent was taken explaining the purchase of the study is purely academic, and how it could help this industry. However, their signed consent in the questionnaire was taken.

### Measurement instrument

Based on the previous studies, the survey instruments were developed. On a 5-point Likert scale, the items were scored from strongly disagree = 1 to strongly agree = 5. Three items each for food quality (FQ), convenience (CON), self-identification (SI), and novelty seeking (NS) scales were adapted from Liew et al. (21), Moon et al. (38), Bîlbîie et al. (100), and Xue et al. (23), respectively. For example, convenience is measured by the questions like “ordering fast food online rather than dining in can save time and money” and self-Identification as a healthy eater is measured by the instrument like “I consider myself to be someone who is worried about the effects of what I eat on my health.” The instrument for food quality measurement was “fast food appeals to me because of its delectable flavor” and for novelty seeking was “novelty and change in my daily routine are the things I like to experience.” Three-item each for cognitive attitude (CA), affective attitude (AA) and subjective norms (SN) were also culled from Moon et al. (38). Cognitive attitude and affective attitude measurement are featured by “during the COVID- 19, buying fast food online are helpful” and “buying fast food online are delightful.” Lastly, the three items for measuring purchase intention (PI) were adapted from Lee et al. (9) measured by “If I get hungry, I plan to order fast food online.”

### Data analysis procedures

To test the theoretical framework, the empirical data was analyzed with the aid of statistical software, such as SPSS 25, MS-Excel, and AMOS versions 21. Construct validity, convergent validity, discriminant validity, and questionnaire reliability were assessed to ensure the accuracy and effectiveness of the questionnaire. The AMOS 21.0 software suite was used to test the links between the postulated components in the structural model. A confirmatory factor analysis (CFA) was utilized in the first stage of Anderson and Gerbing's (101) SEM approach to testing the validity and reliability of the measurement model. Regression analyses were used in phase two of the full structural model to evaluate the overall fitness and postulated links via AMO software.

## Findings of the study

### Demographic profile

The following are the demographics of those surveyed: Seventy-three percent of the participants were men, while 26% were women. All of the responders were between the ages of 26 and 41, indicating that they were youthful. Of the 325 respondents, 64.61 percent had a college degree, followed by 70 (21.53%) and 45 (13.84) respondents with a high school diploma. Also, 30 (9.53%) service holders and 25 (7.69%) entrepreneurs, and more than three third (83.07%) are students among total participated in the survey.

### Data screening and normality

Data screening tests were conducted again before data analysis to account for any inaccuracies in the data gathered online, and no missing or erroneous values were found. The mean of all the numbers that were not outliers were considered for the analysis (102). According to Tabachnicket al. (103), the values of skewness and kurtosis were within the recommended limit (±1 to ±3). The multicollinearity of the independent variables was determined using the tolerance level and Variance Inflation Factors (VIFs) (104). The tolerance values ranged from 0.338 to 0.647 for the first-order variables' VIF values, indicating that none of the variables in **Table 3** exhibit multicollinearity.

#### Measurement model

Next, we followed Anderson and Gerbing's (101) two-step technique, in which the measurement model was first assessed for the reliability and validity of the first and second-order model, and then the structural model was examined for the hypothesis. Latent variables must be validated and proven to be reliable when conducting second-order CFAs. The next part goes in-depth about the reliability and validity of the first-order construct.

#### Common method bias testing

Tests for common method bias were done using Harman's (105) single-factor analysis, which is based on the exploratory factor analysis method. The result showed that only 34.42 percent of the variation was due to a single component, indicating the absence of a typical method bias.

### First-order measurement model

Eight first-order latent variables and twenty-four observed variables were used in the specification search for the Confirmatory Factor Analysis (CFA). Model evaluation was carried out using Maximum Likelihood Estimation (MLE). The latent variables include convenience (CON), food quality (FQ), Novelty Seeking (NS), Subjective Norms (SN), Self-identification (SI), Cognitive attitude (CA), Affective attitude (AA), and Behavioral Intention (BI). The result of the fit indices showed a poor fit in our initial run of CFA, with factor loadings lower than the minimum suggested threshold value (FL ≥ 0.5) (98). Since the factor loadings were <0.5, we deleted them from the new model (98). There was a decent match according to the model fitness (CMIN/DF = 1.521, GFI = 0.955, AGFI = 0.941, CFI = 0.943, RMSEA = 0.056, NFI = 0.929, TLI = 0.932, IFI = 0.943, SRMR = 0.032) (**Table 4**). We also looked at the reliability, convergent validity, and discriminant validity of the measures of constructs employed in the proposed model, which was part of the measurement model analysis process.

Cronbach's alpha (CA) and Composite Reliability (CR) values were used to assess the reliability. Generally, CA ≥ 0.70 (98) and CR ≥ 0.70 (106) is considered as a minimum threshold for assessing the reliability of a construct. In the social sciences, CA and CR levels as low as 0.6 are regarded as acceptable (107, 108). Table 1 shows that the CA and CR values, both of which are above the required minimum threshold of 0.60, demonstrating that all first-order structures are acceptable and satisfactory.

**Table 1:** Cronbach alpha, composite reliability, AVE.

**Constructs**	**Item loading**	**Mean**	**SD**	**Skewness**	**Kurtosis**	**Alpha** **α**	**CR**	**AVE**
**Utilitarian value**								
Convenience (38)		3.290	0.708	0.096	−0.428	0.831	0.851	0.660
CON1: Ordering fast food online rather than dining in can save time and money	0.677							
CON2: Because of their delivery service, I prefer to buy fast food	0.774							
CON3: Shopping on the internet would allow me to shop whenever I wanted	0.961							
Food Quality (21)		3.384	0.642	−0.51	0.248	0.773	0.779	0.541
FQ1: Fast food appeals to me because of its delectable flavor	0.754							
FQ2: On the menu of fast-food meals, there are range of food selections	0.743							
FQ3: The freshness of food is the reason I purchase fast food online	0.708							
**Hedonic value**								
Novelty seeking (23)		3.388	0.778	−0.431	0.227	0.807	0.817	0.598
NS1: Novelty and change in my daily routine are the things I like to experience	0.741							
NS2: Change, variety, and travel, even if it involves some danger, are what I like in a job	0.758							
NS3: New ideas and experiences are what I am continually seeking	0.819							
Subjective Norms (38)		3.287	0.689	0.167	−0.213	0.797	0.797	0.568
SN1: I do buy online fast food with my family in order to socialize	0.779							
SN2: I buy fast food to uphold my status toward my friends	0.705							
SN3: My surrounding people whom I give importance to think that I should buy fast foods online	0.774							
Self-Identification as a Healthy Eater (100)		3.645	0.668	−0.284	0.08	0.831	0.833	0.625
SI1: I consider myself to be a healthy eater	0.752							
SI2: I consider myself to be someone who is concerned about healthy eating	0.788							
SI3: I consider myself to be someone who is worried about the effects of what I eat on my health	0.830							
Cognitive Attitude (38)		3.330	0.815	−0.192	0.13	0.858	0.843	0.643
During the COVID- 19, buying fast food online are: CA1: Effectiv.	0.755							
CA2: Helpful	0.829							
CA3: Practical	0.819							
Affective Attitude (38)		3.415	0.808	−0.348	−0.305	0.874	0.853	0.660
Buying fast food online are: AA1: Fun	0.815							
AA2: Exciting	0.785							
AA3: Delightful	0.836							
Buying Intention (9)		3.404	0.786	−0.211	−0.225	0.900	0.900	0.750
BI1: If I get hungry, I plan to order fast food online	0.851							
BI2: In the future, I plan to continue buying fast food online	0.880							
BI3: In the future, I plan to buy fast food online frequently	0.866							

Authors' calculation.

Furthermore, the measurement model was evaluated using first-order construct validity. In other words, all of the first-order variables were examined for construct validity, which included looking at their convergent and divergent/discriminant validity. AVE and factor loadings were utilized to demonstrate convergent validity. The AVE value of more than 0.5 for all constructs is indicative of convergent validity (106). All first-order constructs have AVE values that meet or exceed the 0.50 threshold, indicating that they have convergent validity. Significant factor loadings of 0.5 for all measurement items are indicated as further proof of convergent validity (109). The fact that all factor loadings were significant and higher than the required threshold confirms the convergent validity (Table 1). Moreover, The Fornell-Larcker criterion and the Heterotrait-Monotraits (HTMT) ratio were used to determine the constructs' discriminant validity. In this case, the square root of a construct's AVE score must be bigger than its maximum correlation with any other construct in the model (106). The findings of this study (see Table 2) satisfy the discriminant validity.

**Table 2:** Discriminant validity of the first-order construct.

**Variables**	**CON**	**FQ**	**NS**	**SN**	**SI**	**CA**	**AA**	**BI**
Convenience	**0.812**							
Food quality	0.411^**^	**0.736**						
Novelty seeking	0.424^**^	0.395^**^	**0.773**					
Subjective norms	0.720^**^	0.525^**^	0.462^**^	**0.754**				
Self-identification	0.453^**^	0.419^**^	0.597^**^	0.441^**^	**0.791**			
Cognitive attitude	0.539^**^	0.481^**^	0.651^**^	0.632^**^	0.551^**^	**0.802**		
Affective attitude	0.539^**^	0.503^**^	0.657^**^	0.546^**^	0.663^**^	0.723^**^	**0.812**	
Buying Intention	0.472^**^	0.500^**^	0.535^**^	0.650^**^	0.470^**^	0.720^**^	0.732^**^	**0.866**

In the Table, bold elements, the square root of AVE.

** Correlation is significant at the 0.01 level (2-tailed).

Similarly, the HTMT, which is linked to a dissipated construct score, assesses the relationship between the constructs (Table 3). This study further shows the absence of discriminant validity problems based on the threshold value of <0.9 (110). Overall, the study found acceptable reliability and validity.

**Table 3:** Discriminant validity of first-order construct using Heterotrait-Monotrait Ratio (HTMT).

**Variables**	**CON**	**FQ**	**NS**	**SN**	**SI**	**CA**	**AA**	**BI**	**Tolerance**	**VIF**	***R* ^2^**
Convenience	–								0.395	2.530	0.28
Food quality	0.512	–							0.647	1.545	0.74
Novelty seeking	0.512	0.491	–						0.470	2.125	0.68
Subjective norms	0.820	0.667	0.569	–					0.328	3.050	0.50
Self-identification	0.538	0.521	0.726	0.537	–				0.502	1.991	-
Cognitive attitude	0.633	0.584	0.773	0.764	0.651	–			0.360	2.777	0.85
Affective attitude	0.628	0.609	0.776	0.654	0.620	0.834	–		0.338	2.955	0.77
Buying intention	0.541	0.593	0.622	0.766	0.544	0.818	0.828	–			0.79

The coefficient of determination (*R*^2^) of the endogenous variable may be used to measure the model's explanatory power (111). The *R*^2^ value of more than 0.26 indicates a strong explanatory power, while <0.13 indicates otherwise. Mild power is defined as any value that falls in between these two categories (112). The model of this study is considered to have a high degree of explanatory capability, since all of the endogenous values found in this study satisfy the analysis criteria according to Falk and Miller (113) (Table 3).

### Second-order measurement model

**Figure 2:**
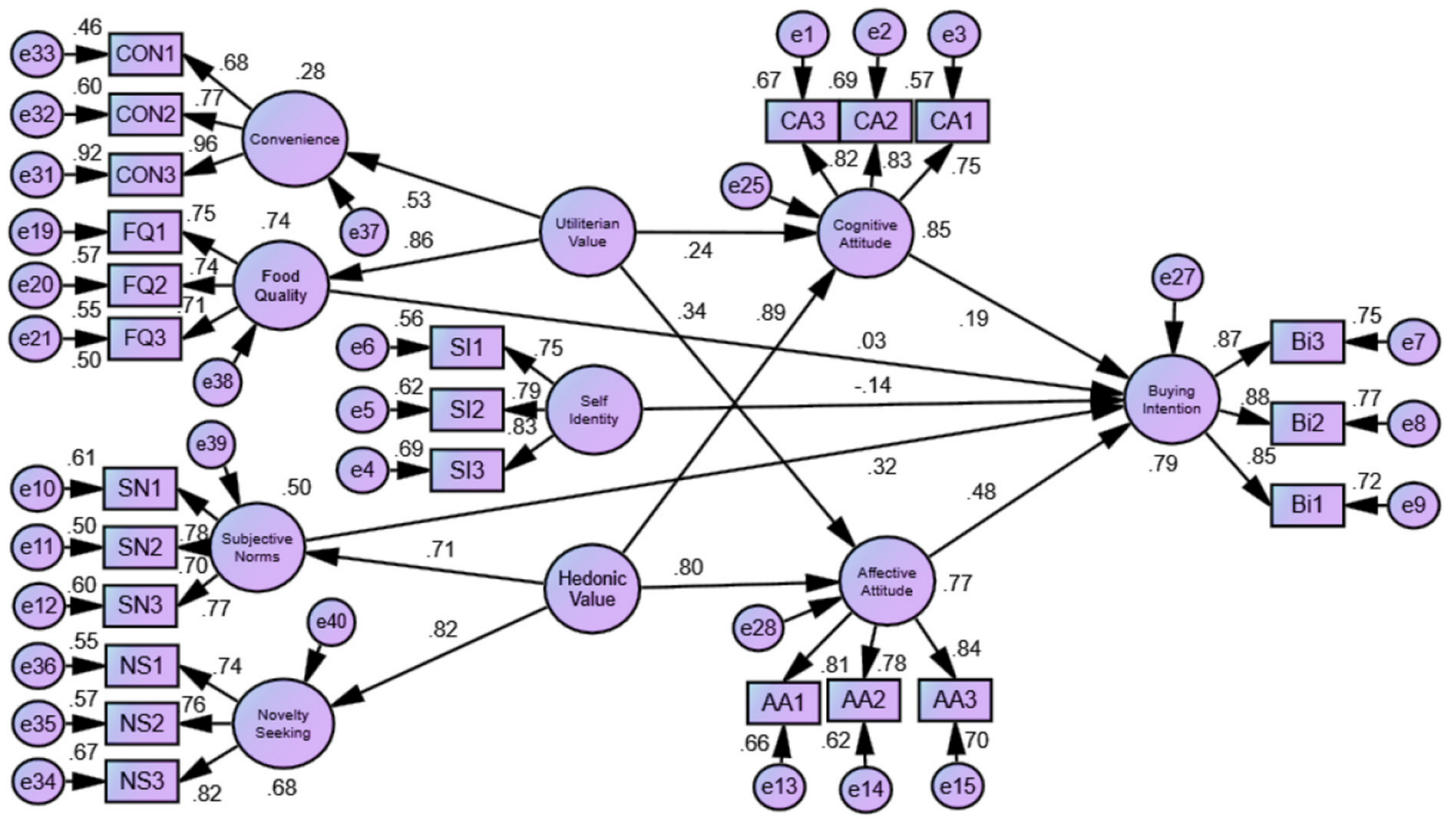
Structural model.

The repeated indicator technique, which measures higher-order constructs using items from all of their lower-order constructs, is a typical way to approximate second-order constructs. This approach is more efficient when all of the lower-order structures have the same number of items. When it comes to second-order CFA, the variables utilitarian value (UV) and hedonic value (HV) were considered as higher-order reflective constructs, whereas the variables cognitive attitude (CA), affective attitude (AA), and purchasing intents (BI) were identified as first-order constructs. As illustrated in Figure 2, lower-order constructs of utilitarian values (UV) were represented as convenience (CON) and food quality (FQ), while novelty seeking (NS) and subjective norms (SN) were treated as hedonic values (HV).

Various items had factor loadings less than minimum recommended threshold value (0.5) and fit indices with poor fit, resulting in our removal of items with factor loadings of <0.5 during re-specification (97). Consequently, the model fit indices demonstrated a satisfactory fit for the model: CMIN/DF = 2.116, GFI = 0.932, AGFI = 0.919, CFI = 0.934, RMSEA = 0.058, NFI = 0.919, TLI = 0.917, IFI = 0.924, and SRMR=0.036 (Table 4). Finally, all path coefficients of the second-order reflecting constructs on the first-order constructs are significant (*p* < 0.01) and surpass 0.50 for utilitarian values (UV) and 0.71 for hedonic values (HV).

**Table 4:** Results of CFA and structural model with standards.

**Fit indices**	**Measurement model for CF**		**Meas. values for structural model**	**Standards with sources**	
	**1st order**	**2nd order**		
χ2/df	1.521	2.116	2.232	<3	(114)
IFI	0.943	0.924	0.912	>0.900	(115)
NFI	0.929	0.919	0.907	>0.900	(115)
CFI	0.943	0.934	0.923	>0.900	(116)
GFI	0.955	0.932	0.921	>0.900	(115)
AGFI	0.941	0.919	0.911	>0.900	(117)
TLI	0.932	0.917	0.905	≥0.90	(118)
SRMR	0.032	0.036	0.047	<0.080	(115)
RMSEA	0.056	0.067	0.058	<0.080	(107, 118)

Source:Authors' calculation.

The reliability of higher-order constructs was investigated using Composite Reliability (CR) and Average Variance Extracted (AVE) from higher-order variables. Two-order constructs had CR scores of 0.733 and 0.766, which are significantly over the 0.70 threshold level for second-order constructs (119). In Table 5, the AVE values of hedonic and utilitarian values are 0.583 and 0.532, respectively, which are both substantially over the minimum acceptance level of 0.50, indicating trustworthy reflective second-order structures (119).

**Table 5:** Discriminant validity discriminant validity of the second-order construct.

**Variables**	**CR**	**AVE**	**BI**	**AA**	**SI**	**UV**	**CA**	**HV**
Buying intention	0.901	0.752	0.867					
Affective attitude	0.875	0.700	0.830	0.837				
Self-identity	0.833	0.624	0.543	0.773	0.790			
Utilitarian value	0.766	0.532	0.629	0.622	0.694	0.708		
Cognitive attitude	0.862	0.675	0.810	0.817	0.650	0.610	0.822	
Hedonic value	0.733	0.583	0.690	0.767	0.753	0.625	0.744	0.764

Source: Authors' calculation.

Finally, the correlation matrix (Table 5) was used to investigate the discriminant validity of first-order (cognitive attitude, affective attitude, and purchase intents) and second-order (utilitarian values and hedonic values) components. Comparing the square root of AVE values with inter-construct correlations, which should be greater than the shared variance between two constructs, is the primary method for determining discriminant validity. This requirement is met by the model's constructs (89). All of the constructs were below the required threshold value of 1.00, suggesting that the discriminant validity of second-order constructs had been reached, as indicated in Table 5.

## Structural model and discussions

Furthermore, we employed the most often used fit indices to assess the suitability of the theoretical model for the data; the computed value of 2/df (chi-square/degree of freedom) is 2.232, which is within the suggested range (2/df ratio of 3 or less). Table 5 shows that the incremental fit indices are >0.90, with CFI = 0.923, GFI = 0.921, AGFI = 0.911, NFI = 0.907, IFI = 0.912, and TLI = 0.905. Furthermore, the RMSEA = 0.058 value is <0.08, indicating a good fit (Table 4). Hence, the goodness-of-fit statistics show that the whole model fits well.

The path coefficients were also assessed to support the hypotheses, as recommended by Hair (120), and the results are reported in Table 6. The H1 projected that customers' judgments of utilitarian values (food quality and convenience) and their cognitive attitude regarding online fast-food purchasing are linked. The study's findings revealed a statistically significant positive link between utilitarian values and cognitive attitude toward online fast-food purchasing (β = 0.52; *p* < 0.05). Arguments supporting this conclusion can be found in studies (9, 38, 72, 121, 122) that show utilitarian values as having a major impact on consumer attitudes toward online fast food purchases. Consumers who value specific food quality, ease of purchase, and a user-friendly internet interface are more likely to exhibit a favorable rational attitude toward online fast-food purchases.

**Table 6:** Structural model and hypothesis testing result.

**Hypotheses**	**STD Beta**	**STD error**	***t*-values**	***P*-values**	**Significance (*p* < 0.05)**
H1: UV → CA	0.240	0.107	3.703^***^	0.000	Supported
H2: UV → AA	0.345	0.125	4.684^***^	0.000	Supported
H3: FQ → BI	0.029	0.066	0.660	0.509	Not Supported
H4: HV → CA	0.888	0.154	8.825^***^	0.000	Supported
H5: HV → AA	0.804	0.141	9.001^***^	0.000	Supported
H6: SN → BI	0.316	0.075	5.129^***^	0.000	Supported
H7: CA → BI	0.186	0.097	2.160^**^	0.031	Supported
H8: AA → BI	0.484	0.095	5.596^***^	0.000	Supported
H9: SI → BI	0–0.135	0.048	−3.454^***^	0.000	Supported

** Significant at 5% level,

*** Significant at 1% level.

The second hypothesis (H2) proposed a link between consumers' perceptions of utilitarian value (food quality and convenience) and their affective attitude toward purchasing fast food online. The findings revealed a strong positive connection between utilitarian value and emotional attitude toward online fast-food purchases (β= 0.345; *p* < 0.00). Past researchers have also discovered a link between utilitarian values and attitudes toward online fast food purchases (9, 38, 72, 122, 123). Our findings imply that consumers who are motivated by utilitarian/functional considerations when shopping for products such as fast food are more prone to make sensational judgments about online fast-food purchases. Consumers who place a premium on food quality, ease of purchase, and a user-friendly website interface are more likely to have a positive emotional appeal toward online fast-food purchases.

The third hypothesis (H3) postulated a positive influence of FQ on BI, which was not supported by the empirical test (H3; β = 0.029, *t* = 0.660, *p* = 0.509). This finding contrasts with the findings of some earlier research studies (22), which reported a strong impact of FQ on green product-purchasing intention. As a result, we concluded that consumers' perceptions of food quality are not directly relevant to fast food purchase intentions. Customers can easily assess the product quality in case of a physical visit to the restaurant and build a perception about product quality. For online fast food buying, this is missing. Probably within a hard lockdown situation, customers might not give that much priority to product quality. Other reasons may be that it forms the utilitarian values and influences intention instead of being direct relations.

The fourth hypothesis (H4) proposed a relationship between customers' perception of hedonic values (subjective norms and novelty-seeking) and their cognitive attitudes toward online fast-food purchases. The findings revealed a positive and significant association between hedonic values (HV) and customers' cognitive attitude (AA) toward online fast-food purchases (β= 0.888; *p* < 0.00). Previous researches have shown that shopping enjoyments are very important and can significantly influence attitude toward online fast food buying (9, 38, 124). Consumers who derive happiness and enjoyment from their success in finding low-cost products, and sharing their positive shopping experience (through a social media platform such as Facebook) with others are more logically inclined to shop online.

The fifth hypothesis (H5) suggested a link between customers' perceptions of hedonic values (subjective norms and novelty-seeking) and their affective attitudes toward online fast-food purchases. The study's findings revealed a favorable link between hedonic values (HD) and affective attitude (AA) toward online fast-food purchases (β = 0.804; *p* < 0.00). The findings are consistent with earlier research (9, 38, 72, 121, 122, 125), where similar relationships were found between hedonic attributes and attitudes toward online fast food buying. Hence, our findings suggest that consumers who derive pleasure, enjoyment, and satisfaction from their success in obtaining low-cost fast-food products and sharing their pleasant shopping experience with others (via a social media site like Facebook) are more psychologically motivated to shop online. Furthermore, the sixth hypothesis (H6; β = 0.316, *t* = 5.129, *p* = 0.00) was confirmed, indicating that SN has a favorable effect on BI. This backed up the findings of recent studies (22), which revealed that Bangladeshi customers are inclined to acquire fast food products if their colleagues and family members recommend them.

The seventh hypothesis (H7) proposed a connection between customer cognition and intent to buy fast food online. This study discovered a positive and significant association between cognitive attitude (CA) and online fast-food purchase intention (β = 0.70; *p* < 0.05). Similar findings has been reported in numerous studies (38, 41, 91, 100). The findings imply that consumers' cognitive attitudes are essential in predicting their desire to buy fast food online and that customers are more involved in cognitive judgments when making their online purchase decision (β = 0.186; *p* < 0.031). Several prior research have suggested a similar link (126). Consumers who analyze online-buying activities emotionally are more likely to form purchase intentions, according to our findings. Our data also show that both variables of attitude have an impact on online fast-food purchase intent. Furthermore, the data suggest that customers' online purchase intentions for fast food are more closely linked to cognitive than emotive judgments. In other words, when making an internet purchase, consumers depend more on their cognitive judgments than their emotional judgments.

Moreover, the study's findings confirmed the H8 that a positive association exists between consumers' affective attitude (H8; β = 0.484, *t* = 5.596, *p* = 0.00) and behavioral intention, which is consistent with previous research (38, 41, 100). The final hypothesized relationship (H9) was found to be negative (H9; β = −0.135, *t* = −3.454, *p* = 0.00), confirming that SI and BI have a negative association. These findings support prior studies (100), which show that the higher a person's self-identity as a healthy eater, the less likely they are to buy fast food online.

## Conclusion and policy implications

The purpose of the study was to determine the factors of fast food-buying intention among Bangladeshi Millennials. The study revealed that convenience and food quality forms utilitarian values, while subjective norms and novelty-seeking forms hedonic values. Also, the utilitarian and hedonic value affects both the cognitive and affective attitude significantly. The cognitive and affective attitude, self-identity, and subjective norms affect behavioral intention, as opposed to food quality. Nonetheless, the affective attitude was found to have stronger predictability of behavioral intention than the other factors, and the model's explanatory power is also appeared as high. Therefore, the major theoretical and practical implications are further discussed.

### Implications of the study

#### Theoretical implications

This study, which undertakes a fresh approach to predict customers' purchase intentions, avail four key contributions to the literature on online retailing: First and foremost, this study provides a sound theoretical framework by modifying the VAB model, which is lacking in earlier studies. Using the higher-order pattern of predicting purchase intentions, the operationalization of the VAB model sheds light on how to better forecast purchase intentions in the online retailing literature. Second, the study investigated the millennial consumer's online fast-food buying intention, which is not studied earlier in the Bangladeshi context. Thus, it offers a significant contribution to academia and literature in this field.

Third, this research contributes to a better understanding of the factors of customer purchase intentions by incorporating variables of attitude into the structural model of the consumer. As part of an effort to better understand how attitude affects individual behavior, previous studies (42, 127, 128) have debated the existence of two types of attitude: affective and cognitive. This is necessary to know how attitude affects individual behavior. The most important findings of this research revealed that attitudes toward cognition and emotion are related to intention in various ways and depend on diverse circumstances. The study's inclusion of cognitive and affective attitudes contributes to a better understanding of consumers' rational and emotional appraisals of their intents to acquire fast food from websites.

Fourth, distinct underlying proxies of utilitarian and hedonic traits are explored in terms of their separate impacts rather than the combined effects of the underlying attributes. The study achieves a higher degree of abstraction by employing utilitarian and hedonic qualities as reflective second-order constructs in the first place. Fifth ***and last***, this article investigates the factors of online-purchasing intention throughout the quarantine period in order to assist restaurants in planning for both current restrictions as well as a post-vaccination period. Although there is a chance of reducing online fast food buying intention after the post-vaccination period, the trend of online buying will regain gradually as rapid urbanization is occurring and the participation of women in the workplace is increases, particularly in Dhaka city. The restaurant's authority can sustain its online selling by taking audience-based (targeting generation Y consumers) promotional efforts with the result of this study supplied.

#### Practical implications

The findings of this study have a wide range of applications in the world of online retail. ***First***, there are a few things that online merchants should keep in mind while designing their shopping websites. The website interface of online shops should be simple and user-friendly. Consumers are more likely to make purchases if the website is easy to use because they need to invest money. Online shoppers place a high value on getting all the information they need on the products they intend to purchase. Consumers' perceptions of a product's performance are more likely to be clarified if the manufacturer provides comprehensive product information. In addition, customers are more inclined to undertake a functional assessment of a product when presented with comprehensive information. Online shoppers place a high value on the perceived financial benefits that come with a product when they take advantage of promotional offers. In addition to brick-and-mortar stores, retailers should look to online shopping platforms that allow customers to shop when and where they want. Online businesses can obtain a competitive advantage over their rivals by incorporating these functional qualities into their online shopping websites.

Second, although it is less important than utilitarian factors, hedonic aspects of e-commerce sites should not be ignored by companies selling online. Hedonic factors influence customers' decisions to buy online. Many shoppers find shopping to be an enjoyable experience and derive pleasure and amusement from it. To attract more clients, online shops should provide social engagement, discounted offers and pricing, and role-playing on their sites.

Third, the fast-food sector must devote more resources to research in order to better understand customer behavior and beliefs. Managers must have a deeper understanding of the implications of a customer's purchasing intention while using certain channels. We should study who these external factors are so that we can craft methods that may influence the consumer's subjective norm and buy intention. Fast-food chains that eye growth must devote more resources to promoting food values, good emotions, eating attitudes, brand attitudes, and other subjective standards.

### Limitations and future research direction

The present study, which analyzed purchasing behavior by assessing intention, has certain limitations in terms of generalizability. Hence, a future study that incorporates actual behavior into the model to understand young people's purchasing behavior may be able to provide more detailed results. Furthermore, although a substantial number of research studies have been published on fast-food consumption among teens, less is known about fast-food consumption among older age groups. This study included those 26–41 years old consumers and should not be generalized beyond this group. Also, this study was only conducted in the context of Bangladesh, not other geographical locations. Further research is required to close the gap, with a particular emphasis on the purchasing habits of older individuals who eat fast food all over the world. As research on fast-food consumption in Bangladesh is essentially non-existent, our findings cannot be compared to local theoretical foundations or earlier findings. Although our sample is supposed to include people from varying localities, the bulk of our respondents were Dhaka students between the ages of 18 and 35 who were selected using convenience sampling. A study conducted on a representative sample of people from various places may provide researchers with deeper insight into what motivates this age group's online fast-food consumption. The study is also cross-sectional because it only looked at data from one moment in time on a survey. A cross-sectional design study does not capture causal inferences and, therefore, does not capture factors that influence something, but rather factors associated with a phenomenon. Thus, it may be expanded by conducting more experiments in other settings or adopting a longitudinal strategy and conducting more in-person interviews.

## Data availability statement

The raw data supporting the conclusions of this article will be made available by the authors, without undue reservation.

## Ethics statement

Ethical review and approval were waived for this study due to the fact that there is no institutional review board or committee in Bangladesh. The study was conducted as per the guidelines of the Declaration of Helsinki.

## Author contributions

Conceptualization: CY. Data curation: AS, MM, and MH. Funding acquisition: QD. Investigation: MM and AI. Methodology: CY and AS. Project administration: ZG-W and MM. Resources: ZG-W, MM, MH, and AI. Software: AS. Validation: AS and AI. Visualization: MH. Writing—original draft: CY, AS, and MM. Writing—review and editing: CY, QD, MH, ZG-W, and AI. All authors contributed to the article and approved the submitted version.

## Funding

This study was funded by the Key Project of the National Social Science Foundation of China (Grant No. 20AJY015), the Fundamental Research Funds for the Central Universities (Grant No. 300102341667), and the Innovation Capability Support Program of Shaanxi (Grant No. 2022KRM145).

## Conflict of interest

The authors declare that the research was conducted in the absence of any commercial or financial relationships that could be construed as a potential conflict of interest.

## Publisher's note

All claims expressed in this article are solely those of the authors and do not necessarily represent those of their affiliated organizations, or those of the publisher, the editors and the reviewers. Any product that may be evaluated in this article, or claim that may be made by its manufacturer, is not guaranteed or endorsed by the publisher.
